# PD68 Access To Medications For Chronic Degenerative And Rare Diseases In The Brazilian Public Health System: 2012 to 2023

**DOI:** 10.1017/S0266462325102006

**Published:** 2025-12-29

**Authors:** Amanda Oliveira Lyrio, Ana Carolina de Freitas Lopes, Luciene Bonan

## Abstract

**Introduction:**

Medications for chronic degenerative and rare diseases are provided by the Brazilian Unified Health System (SUS) after health technology assessment analysis conducted by the National Committee for Health Technology Incorporation (CONITEC). Access to these medicines at an outpatient level occurs through the Specialized Component of Pharmaceutical Assistance (CEAF). This study analyzed the number of people who had access to medicines included in the CEAF from 2012 to 2023.

**Methods:**

Administrative and national dispensing data were extracted from the Health Intelligence Open Situation Platform (SABEIS), covering the period from January 2012 to December 2023, with data updated in August 2024. SABEIS consolidates open data from Ambulatory Information System (SIA/SUS), providing anonymized, individualized data. The annual number of users receiving medications from the CEAF was evaluated over this period, starting from the establishment of CONITEC in 2012.

**Results:**

Findings indicated a continuous growth in CEAF users between 2012 and 2023, with a total of 10,969,433 users and 84 different clinical conditions. The number of users grew from 1,991,828 in 2012 to 3,783,957 in 2023, reflecting an approximate 90 percent increase over 12 years and an average annual increase of 163,000 new users. During 2019 and 2020, there was stabilization, with only 11,332 new users, potentially due to the impact of COVID-19 on distribution and access. From 2021 onward, growth accelerated, with annual additions of 200,000 to 350,000 users, suggesting a recovery and expansion in treatment coverage.

**Conclusions:**

The data highlighted an increasing demand for specialized treatments and the impact of CONITEC’s decisions on expanding access to high-cost treatments for chronic-degenerative and rare diseases in the Brazilian SUS.

